# Reusable period products: use and perceptions among young people in Victoria, Australia

**DOI:** 10.1186/s12905-023-02197-3

**Published:** 2023-03-11

**Authors:** Caitlin Ramsay, Julie Hennegan, Caitlin H. Douglass, Sarah Eddy, Alexandra Head, Megan S. C. Lim

**Affiliations:** 1 Maternal, Child and Adolescent Health Program, Burnet Institute, Melbourne, VIC Australia; 2 Melbourne School of Population and Global Health, University of Melbourne, Melbourne, Australia; 3 School of Population Health and Preventive Medicine, Monash University, Melbourne, Australia

**Keywords:** Menstrual health, Menstrual hygiene, Adolescent girls, Environmental sustainability, Health policy, Reproductive health, Women’s health

## Abstract

**Background:**

Reusable menstrual products have expanded the choices available for menstrual care and can offer long-term cost and environmental benefits. Yet, in high-income settings, efforts to support period product access focus on disposable products. There is limited research to understand young people’s product use and preferences in Australia.

**Methods:**

Quantitative and open-text qualitative data were collected through an annual cross-sectional survey of young people (aged 15–29) in Victoria, Australia. The convenience sample was recruited through targeted social media advertisements. Young people who reported menstruating in the past 6 months (n = 596) were asked questions about their menstrual product use, use of reusable materials, product priorities and preferences.

**Results:**

Among participants, 37% had used a reusable product during their last menstrual period (24% period underwear, 17% menstrual cup, 5% reusable pads), and a further 11% had tried using a reusable product in the past. Reusable product use was associated with older age (age 25–29 PR = 3.35 95%CI = 2.09–5.37), being born in Australia (PR = 1.74 95%CI = 1.05–2.87), and having greater discretionary income (PR = 1.53 95%CI = 1.01–2.32). Participants nominated comfort, protection from leakage and environmental sustainability as the most important features of menstrual products, followed by cost. Overall, 37% of participants reported not having enough information about reusable products. Having enough information was less common among younger participants (age 25–29 PR = 1.42 95%CI = 1.20–1.68) and high school students (PR = 0.68 95%CI = 0.52–0.88). Respondents highlighted the need for earlier and better information, challenges navigating the upfront cost and availability of reusables, positive experiences with reusables, and challenges for use, including cleaning reusables and changing them outside the home.

**Conclusions:**

Many young people are using reusable products, with environmental impacts an important motivator. Educators should incorporate better menstrual care information in puberty education and advocates should raise awareness of how bathroom facilities may support product choice.

**Supplementary Information:**

The online version contains supplementary material available at 10.1186/s12905-023-02197-3.

## Background

Women, adolescent girls, and all people who menstruate need access to sufficient, safe, and comfortable mechanisms to collect menstrual bleeding and to make informed decisions about their self-care and product use, as part of their menstrual health [1–4]. In Australia, people regularly purchase commercial products such as disposable pads and tampons, with an estimated 300 million tampons and 500 million disposable pads sold each year [5]. Disposable products have a significant environmental cost. In the UK, menstrual products generate an estimated 28,114 tonnes of waste annually [6]. However, studies have reported that people have mixed awareness of the environmental consequences of disposable products [7].

Reusable menstrual products such as period underwear, reusable pads and menstrual cups are alternatives to disposables and may offer greater environmental and cost sustainability. [8–10] A recent environmental assessment found that over one year, the environmental impact of a menstrual cup was equal to less than 1.5% of the environmental impact of a disposable product, at only 10% of the cost of disposables [8]. Another estimate suggested use of menstrual cups comprised only 7% of the cost and 6% of the plastic waste of tampons [9]. For people menstruating in high-income countries, using a reusable pad was estimated to save US$205 and the use of 1,300 single use tampons [10]. Recent systematic reviews have found that both menstrual cups and reusable pads are safe and effective products [9, 10]. Despite these benefits, an audit of menstrual education websites globally found only 30% mentioned menstrual cups and 22% reusable pads [9].

Limited research has documented the uptake and acceptability of reusable products and the barriers and facilitators to use, particularly in high-income countries [7, 9–13]. A large survey of 8,658 nurses in South Korea found that most participants used disposable menstrual products (89%) and few reported using cloth menstrual pads (5%) or menstrual cups (2%) [13]. Participants chose to use disposable products because they were convenient to change and easy to discard. In contrast, participants used menstrual cups for their comfort, eco-friendliness and for health reasons. A large mixed methods study of women and people who menstruate in Spain (aged 18–55) in 2021 found 55% had used reusable menstrual products including menstrual cups (48%), reusable pads (15%) and menstrual underwear (9%). Use of reusable products differed by age, place of birth, gender identity, completion of education, and wealth. [14]. Qualitative interviews with 34 of these participants perceived menstrual cups as clean, comfortable, environmentally-friendly, and healthier in comparison to disposable products. However, participants also noted barriers to using menstrual cups including physical pain after prolonged use and inadequate space for cleaning and management, particularly when around other people at home [14].

Recent efforts to address “period poverty” in young people, such as the provision of free menstrual products in schools [15], have focused almost exclusively on disposable products. In Australia, there are limited data capturing young people’s use or perceptions of reusable products. One study of university students in 2020 found 65% of respondents mostly used pads, 24% tampons, 8% a menstrual cup at 2% period underwear [16]. Another online survey of females in 2020 found 33% reported difficulties accessing period products during the COVID-19 pandemic, this was more common among younger women [17]. Neither study investigated perceptions or experiences of reusable products. It is unclear if current efforts to provide free menstrual products reflect the preferences of young people, or if alternative strategies may support the use of more environmentally and cost-effective products. Thus, the present study aimed to:

1. Describe the use of reusable menstrual products among young people in Victoria and explore demographic differences in their use
2. Understand young people’s product priorities and perceptions of reusable products
3. Describe young people’s access to information about reusable products

## Methods

### Data collection

This study used data from the Burnet Institute’s Sex, Drugs, and Rock’n’Roll (SDRR) study, an annual online cross-sectional survey with a convenience sample of young people from Victoria, Australia [18]. The questionnaire was developed by the authors and includes 134 total questions on various health topics, including 23 questions on menstrual products. All materials are in English language.

The Alfred Health Research Ethics Committee approved the study (project number 326/08).

### Setting

Recruitment was conducted online between April and June 2021, mainly via social media platforms such as Facebook and Instagram. The survey was distributed through paid advertisements and individual post sharing. Individuals who completed the survey in past years and provided consent to be contacted for future research were also emailed a link to the survey. Advertisements did not specifically mention menstrual health or menstrual products.

### Participants

Participants were eligible to complete the survey if they were aged 15–29 years and lived in Victoria, Australia. After clicking on the survey link, participants were provided with a participant information sheet. Once participants provided informed consent, they commenced the survey through the secure Research Electronic Data Capture (REDCap) system [19, 20]. 1001 responses were collected. For this study, the sample was limited to participants who reported having a menstrual period in the last six months (n = 596).

### Variables and measurement

#### Demographic characteristics

Socio-demographic factors assessed included age, gender identity, country of birth, discretionary income, current level of study and remote residence. Remoteness was determined by classifying participants into major city of Australia or regional Australia based on postcodes provided.

#### Product use

Participants were asked to report all the menstrual products or materials they had used during their most recent period as outlined in the Menstrual Practices Questionnaire [21]. In addition, they were asked if they had ever tried using a reusable product. In comparing perceptions of reusable products, participants were grouped into three categories: never users (those who had never used a reusable product), ever users (those who had ever used a reusable product but not during the last period), and current users (those who reported using a reusable product during their last menstrual period).

#### Product preferences and perceptions

From a list of 12 features, participants were asked to nominate the three they considered most important when selecting a menstrual product. The selected top features were compared, along with dichotomous variables reflecting whether each feature was included participants ‘top three’.

#### Perceptions of reusable products

Participants were presented with seven statements about reusable products, of which four were positive (good for the environment, low cost, comfortable, good protection from leakage) and three were negative (difficult to change, unhygienic/dirty/gross, too much effort to clean). They were asked to indicate whether they agreed or disagreed with each statement.

#### Product information

Participants were asked if they agreed or disagreed with the statement “I have enough information about reusable products.”

#### Open-text survey responses

At the end of the set of menstrual health questions, participants were presented with an optional open text question asking: “Is there anything else you’d like to tell us about making menstrual product choices or the use of reusable products?”

### Data analysis

Data were downloaded from REDCap and analysed in Stata 17. Descriptive statistics were used to report the proportion of participants using different products, reusable products, and the features considered most important when considering a menstrual product.

Multinomial logistic regression was used to determine the demographic characteristics associated with reusable product use. Poisson regression with robust error variance (modified Poisson) was used to test differences in the perceptions of reusable products among never, ever, and current users of reusable products. Robust Poisson regression was also used to test associations between demographic characteristics and having sufficient information about reusable products. Chi-squared tests were used to test if features considered most important when considering a menstrual product differed between current, ever, and never users.

Participants’ open text responses reporting if there was “anything else” they would like to share about product choices or reusables was analysed using a conventional qualitative content analysis approach [22, 23]. Following initial familiarisation, a preliminary coding framework was developed, and each response was coded in Microsoft Excel. The framework was then adapted to incorporate new codes identified inductively. The framework was refined to a final set of categories based on the data and discussion among analysts (JH AH). This reflected a pragmatic approach to qualitative content analysis of the short, but informative, participant responses, with the goal of describing participant needs to inform future research and practice.

## Results

Of the 1001 participants who completed the SDRR survey, 596 reported having a menstrual period in the past six months.

### Menstrual product access and use

**Fig. 1:**
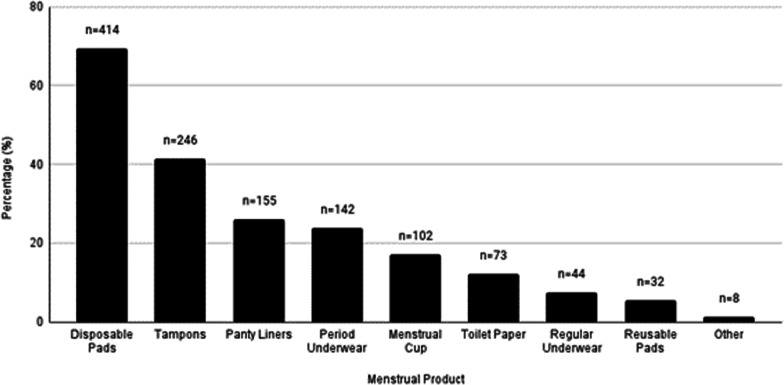
Menstrual products used by the participants in their last menstrual period (n = 595). *Note* Participants could choose multiple options

Figure 1 presents all the menstrual materials used by participants during their last period. Disposable pads were used by most participants (69.5%), followed by tampons (41.3%). A total 222 (37%) of participants had used a reusable menstrual product during their last period, the most common was reusable underwear (23.8%) followed by menstrual cups (17.1%). A further 62 participants (10.6%) reported that they had ever tried using a reusable product but did not report using one during their last period, leaving 310 participants (52.1%) who had never used a reusable product.

Participant demographic characteristics and their association with use of a reusable product are displayed in Table 1. Use of a reusable product during the last period was associated with older age, being born in Australia, and having a higher discretionary income. High school students were less likely to be a current reusable user than tertiary students, while those not currently studying were more likely to be a current reusable user. Having ever used a reusable product was only associated with older age and higher discretionary income. There was no association between reusable product usage and rural residence or gender identity.

**Table 1:** Reusable product use according to demographic characteristics

Demographics (%, n)	Never used a reusable % (n)	Ever used a reusable % (n)	PR (95%CI)^a^	Currently using a reusable % (n)	PR (95%CI)^a^
All participants (n = 595)	52.1 (310)	10.6 (63)		37.3 (222)	
Age (Years)					
15–19 (30.5, 182)	69.2 (126)	6.0 (11)	1.00	24.7 (45)	1.00
20–24 (40.9, 244)	48.6 (118)	11.1 (27)	2.62 (1.24–5.52)	403 (98)	2.33 (1.51–3.59)
25–29 (28.5, 170)	38.8 (66)	14.7 (25)	4.33 (2.01–9.36)	46.5 (79)	3.35 (2.09–5.37)
Missing (n = 0)	(0)	(0)		0	
Area of residence					
Major city (89.2, 445)	50.5 (224)	9.0 (40)	1.00	40.5 (180)	1.00
Regional Australia (10.8, 54)	48.2 (26)	9.3 (5)	1.08 (0.39–2.97)	42.6 (23)	1.10 (0.61–1.99)
Missing (n = 97)	(60)	(18)		(19)	
Country of birth					
Australia (84.3, 494)	49.9 (246)	10.6 (52)	1.34 (0.62–2.87)	39.6 (195)	1.74 (1.05–2.87)
Other (15.7, 92)	62.0 (57)	9.8 (9)	1.00	28.3 (26)	1.00
Missing (n = 10)	(7)	(2)		(1)	
Gender identity					
Female (91.9, 545)	51.7 (281)	10.5 (57)	1.00	37.9 (206)	1.00
Non-female (male, non-binary) (8.1, 48)	56.3 (27)	12.5 (6)	1.10 (0.43–2.77)	31.3 (15)	0.76 (0.39–1.46)
Missing (n = 3)	(2)	(0)		(1)	
Current education level					
High School (14.0, 83)	68.7 (57)	9.6 (8)	0.68 (0.30–1.53)	21.7 (18)	0.47 (0.26–0.83)
Tertiary studies (58.3, 346)	53.0 (183)	11.0 (38)	1.00	35.9 (124)	1.00
Not studying (27.8, 165)	41.2 (68)	10.3 (17)	1.20 (0.64–2.27)	48.5 (80)	1.74 (1.17–2.58)
Missing (n = 2)	(2)	(0)		(0)	
Discretionary income					
< $120 (76.0, 441)	54.7 (241)	9.1 (40)	1.00	36.3 (160)	1.00
≥ $120 (24.0, 139)	41.7 (58)	15.8 (22)	2.29 (1.26–4.14)	42.5 (59)	1.53 (1.01–2.32)
Missing (n = 16)	(11)	(1)		(3)	

^a^Never using a reusable product is the reference category

*PR* prevalence ratio, *CI* confidence interval

### Product perceptions

**Fig. 2:**
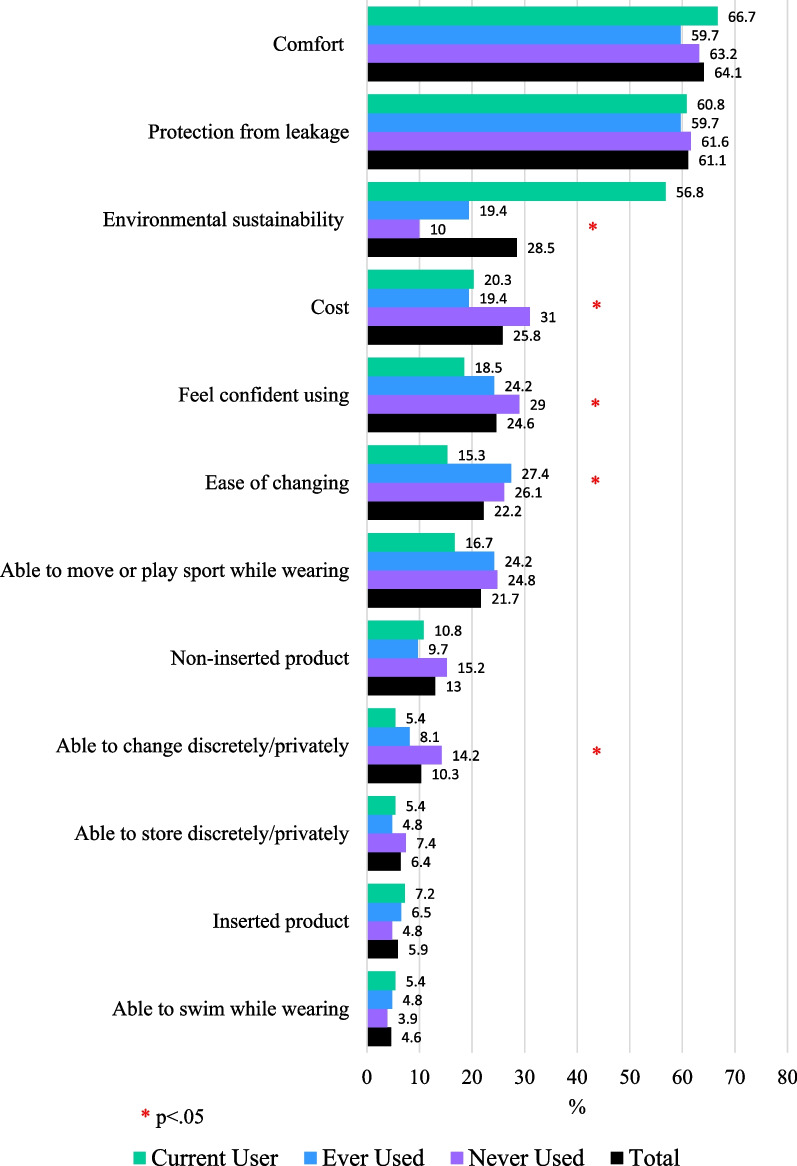
Participants top rated features according to reusable use

When asked to nominate their three most important characteristics of menstrual products, comfort, protection from leakage, and environmental sustainability were endorsed by the greatest proportion of participants, followed by cost, confidence using the materials, ease of changing and ease of mobility when using the product. Figure 2 displays the proportion of participants who included each feature in their three most important features according to their use of reusable materials. When separated by reusable use status, comfort and protection from leakage remained the top two features in every group. The third most important feature differed between groups including cost for participants who had never used reusable products, ease of changing for ever users and environmental sustainability for participants who currently used reusable products.

Table 2 presents participants’ perceptions of reusable products. Current users had more positive perceptions of reusables than those who had never used them. Those who had used reusables, but not during their last period, typically rated reusables more favourably than those who had never used them but less favourably than those using reusables during their last period. Current users of reusable products were more likely than never users to agree that reusables are good for the environment, low cost, comfortable, good protection from leakage, and that they have enough information about reusable products. Current users of reusable products were less likely than never users to agree that reusables are difficult to change outside of the home, too much effort to clean, and are unhygienic/dirty/gross (comparisons presented in Additional file 1: Table S1).

**Table 2:** Proportion of participants agreeing with statements about reusables by reported reusable menstrual product use

Agreement with the statement…	Total %	Never %	Ever %	Current %	Missing (n)
Reusables are good for the environment	92.4	89.7	85.7	98.2	(8)
Reusables are low cost	56.6	49.0	71.4	63.1	(7)
Reusables are comfortable	63.5	48.4	52.4	87.8	(46)
Reusables are good protection from leakage	68.2	58.7	55.6	85.1	(50)
Reusables are difficult to change outside the home	74.6	78.4	71.4	70.3	(22)
Reusables are unhygienic/dirty/gross	20.7	29.7	25.4	6.8	(13)
Reusables are too much effort to clean	47.2	64.8	47.6	22.5	(21)

### Access to information

More than one third of participants reported that they did not have enough information about reusable products. Table 3 presents participants knowledge of reusable products according to demographic characteristics. Among the demographic characteristics included in the survey, younger participants (aged 15–19) and high school students were significantly less likely to report having sufficient information on reusables (47.7% and 55.7%, respectively).

**Table 3:** Information about reusable products by demographic characteristics

Demographics	Has enough information about reusable products %(n)	Does not have enough information about reusable products %(n)	PR (95%CI)
All participants (n = 581)	63.5 (369)	36.5 (212)	
Age			
15–19	52.3 (92)	47.7 (84)	1.00
20–24	64.4 (154)	35.6 (85)	1.23 (1.04–1.46)
25–29	74.1 (123)	25.9 (43)	1.42 (1.20–1.68)
Missing (n)	(0)	(0)	
Area of residence			
Major city	66.7 (291)	33.3 (145)	1.00
Regional Australia	62.3 (33)	37.7 (20)	0.93 (0.74–1.16)
Missing (n)	(45)	(47)	
Country of birth			
Australia	64.9 (313)	35.1 (169)	1.00
Other	58.4 (52)	41.6 (37)	0.90 (0.75–1.09)
Missing (n)	(4)	(6)	
Gender identity			
Female	63.7 (339)	60.9 (28)	1.00
Non-female (male, non-binary)	36.3 (193)	39.1 (18)	0.96 (0.75–1.22)
Missing (n)	(2)	(1)	
Current education level			
High School	44.3 (35)	55.7 (44)	0.68 (0.52–0.88)
Tertiary studies	65.5 (222)	34.5 (117)	1.00
Not studying	69.6 (112)	30.4 (49)	1.06 (0.93–1.21)
Missing (n)	(0)	(2)	
Discretionary income			
< $120	62.3 (268)	37.7 (162)	0.90 (0.79–1.03)
≥ $120	69.3 (95)	30.7 (42)	1.00
Missing (n)	(6)	(8)	

### Qualitative findings

When asked if there was “anything else” they would like to share about product choices or reusables, 97 participants (16%) provided a response. Participants highlighted knowledge and information needs related to reusable products (n = 28), the upfront cost or challenges with accessibility of reusables (n = 27), hesitancy or hygiene concerns related to reusables (n = 13), experiences of discomfort, leakage or difficulties changing reusables outside the home (n = 22), the burden of cleaning reusables (n = 11), positive experiences of comfort and learning to use reusables (n = 16), and challenges related to disorders, bleeding changes, degree of menstrual flow, or contraceptives (n = 9). Seven participants described their desire to try reusables.

#### Knowledge and information

Many respondents described wanting more, or to receive earlier information about reusable products, with some noting they had little awareness of reusable products prior to the survey. Multiple participants reported wishing they were aware of reusables earlier and that information had been provided along with puberty or sexual education in school. Other participants described more detailed informational needs, particularly regarding the use and fit of menstrual cups, including methods of insertion, cleaning, concerns about potential harms or selecting the correct size.

“I would have loved to have learnt more about reusable menstrual products during sex ed at high school – they have totally changed the relationship I have with my period and womanhood”

“…a lot of the information I've encountered about menstrual cups has been very unhelpful - for example, I've seen claims that menstrual cups should work for everyone, which hasn't been the case for me at all. Also, some brands call their smaller sizes for kids or for virgins, which sucks.”

“I wish I knew more about my anatomy/felt more familiar with my body so I could have made this choice earlier on and felt less intimidated”

“There wasn't enough information on the packaging about the different ways to insert the menstrual cup so I watched videos online”

#### Upfront costs and accessibility

Participants noted that reusable products were mostly available online, rather than accessible in stores, making them more difficult to access. A number also noted struggling to find reusable underwear in larger sizes or in a preferred style such as fuller coverage.

“I think reusable menstrual products should be more widely available, especially local stores to avoid having to buy them online”

The upfront cost of both menstrual cups and reusable period underwear was highlighted. While participants noted the potential for savings over the long-term, those on restricted budgets found the upfront cost steep, particularly if they were not confident the product would work for them. Participants noted that multiple pairs of period underwear were needed and that the cost quickly added up.

“Cup is my fave. Not really for me, but I wish there was some way you could try out brands to find a good fit without putting down the $50 each time. Massive deal-breaker for a lot of people I speak to is that they like the cup, but took a while to find a brand which works for them.”

“The initial cost is what puts me off. I understand it would be more cost efficient in the long term, but when living week-to-week, sometimes you can't afford that cost at one time.”

#### Hesitancy and hygiene concerns

Beyond the upfront cost, some participants described concerns about inserting and removing menstrual cups, the hygiene of reusable options, or reusable being inappropriate for varying anatomies or menstrual flow.

“Scared of inserting the product in and not being able to pull it out”

“I find it unhygienic due to the correct way to discard residue in public areas. I think there's a risk to infections and is very awkward to sanitise the product in public wash rooms such as menstrual cups”

#### Discomfort, difficulties changing, and leakages

Participants who had tried reusable products described varied experiences. They described difficulties inserting or removing a menstrual cup, a significant learning curve, or discomfort with the cup inserted. Others described experiencing leaking using reusable products and losing confidence in their performance, or not wanting to invest more funds in finding the right product.

“Took me a year to figure out how to insert a menstrual cup properly.”

“I've used a mensural cup before with both success and failure. Sometimes when I would insert it I could feel it inside me and would have to readjust. I've also experienced leakage and I don't know if that was my fault or the product.”

Most participants in this category mentioned difficulties changing reusables outside the home.

“I tried a menstrual cup and re-usable pads and both products leaked and were very difficult to change during a busy work day.”

“Dude how do I change/empty a cup in public places if I have heavy flow. That just seems like a disaster waiting to happen and no one seems to talk about it”

“Re-usable menstrual products although cost efficient long term, are not fitted for people with very heavy flows and can be uncomfortable to change in work/school environments”

#### Burden of cleaning

Similarly, participants highlighted the burden of cleaning reusable products as a disincentive for use , particularly laundering reusable underwear or pads.

“It's just too hard to wash. You have to soak it in water and then machine wash. It's too much of a hassle.”

“Because of how expensive the reusable period undies are I have only purchased 3 pairs, after wearing one all day, then one at night and one the next day I would have to wash them every day to be able to have a new one for the second night. Sadly, i just don't have the time and so I have to use reusable pads in between.”

A few participants noted feeling pressure to adopt more environmental practices at the cost of inconvenience during their period.

“I do think the marketing can be harsh on vagina owners - like we get enough pressure from society about having a period and not letting it interfere with our everyday lives. Now having a period while trying to 'save the planet' can be a lot of pressure”

#### Comfort and positive learning experiences

Positive experiences of menstrual cups and reusable underwear were described by many participants, noting benefits in comfort and environmental sustainability. Some cup users noted a significant learning curve but reflected positively on learning to use this product and their current experience.

“I got a cup 3 years or so ago. It took me about 18 months to become truly comfortable using it, but now that I am I love it. I think a lot of people get put off by the insertion/cleaning, but it's good to learn how to do it through experience.”

“Menstrual underwear have changed my life. They are so much more comfortable and hygienic.”

“The period underwear has been the best addition to my routine and I look forward to also exploring other products such as moon cups. I think that a lot of people shy away from such products because of the stigma still surrounding periods- whether people like it or not, it's definitely still there!”

#### Desire to try reusable products

While highlighting cost, informational, and access concerns, several respondents also highlighted their desire to try reusable materials.

“Would like to use, need to do more research.”

“I've never really explored reusable menstrual products, but I want to. I don't think I've ever seen any for sale in the supermarket.”

#### Menstrual characteristics

Finally, some participants noted that lighter bleeding or spotting related to menstrual disorders or hormonal contraceptives was not a good fit for a reusable product such as a menstrual cup or noted that heavy bleeding or pain made cleaning reusables too burdensome. Additionally, one respondent commented on the challenge of using reusable products throughout the whole menstrual cycle as the period gradually lightens towards the end of the cycle.

“Quite heavy periods mean I don't have confidence trying reusable menstrual products.”

“So far I have found it difficult to use reusable products throughout the whole cycle as the amount of bleeding starts to lighten, so some products don't feel the most suitable.”

## Discussion

This study describes young people’s use of menstrual products in Victoria, their perceptions of reusable products, access to information, and potential barriers to use.

Overall 47% of participants had ever used a reusable product, similar to survey data from the same time period in Spain finding 51% of respondents aged 18–25 used reusable products [14]. In our sample, reusable period underwear was the most common reusable product used during the last period (24%) followed by menstrual cups (17%). Findings suggest that the experiences of using reusable menstrual products are highly relevant to understanding young people’s menstrual experiences and needs, with almost half of young people in our study already using these products.

When selecting a menstrual product, young people prioritised comfort and effectiveness (protection from leakage). Environmental sustainability was also important, particularly among participants who used reusable products, suggesting that environmental concerns may be a significant motivator for use. Cost, confidence using the product and ease of changing were selected by 20–25% of participants as top priorities. Interestingly, ease of changing was prioritized by fewer current users of reusables but was often selected as a top priority among those who had tried a reusable but did not use one during their last period. Almost all participants perceived reusable products as good for the environment. To promote sustainability in their policies, governments could consider mechanisms for incorporating education and reusable products in school provisions, addressing this initial upfront expense.

Use of reusable menstrual products was associated with older age, being born in Australia, and reporting a higher discretionary income. Additionally, high school students were less likely to use reusable menstrual products than tertiary students. These findings are consistent with participants’ reported access to information about reusable menstrual products, with high school students (56%) and younger participants aged 15–19 (48%) more likely to report they did not have enough information about reusable products. In open-text responses, participants highlighted difficulties accessing enough information about reusable menstrual products, consistent with past audits of information provision [9] and participants suggested to incorporate more comprehensive menstrual product information in school puberty education programs. This finding aligns with a qualitative investigation of menstrual health education in Australian schools, in which students reported a heavy focus on menstrual biology and anatomy with inadequate information about menstrual health more broadly [24]. Greater access to reusable product information and education on how to select and use products such as menstrual cups may increase confidence and enable menstruators to make informed product decisions. Improved visibility and information may also reduce the risks associated with reusable products’ high up-front cost, if users can be more confident that the product will be suitable when purchasing. Beyond information, younger people may also have poorer access to products. As participants noted in open-text responses, many reusable products need to be purchased online and are not readily available in supermarkets and shops.

Many participants perceived reusable products as being difficult to change outside the home (75%), including those who had ever or were currently using reusables (70%). This is consistent with open text comments describing inadequate time, privacy, or concerns about mess when changing reusable products. Further research should explore in more detail the effectiveness of changes to bathroom facilities for supporting menstrual health. In an online survey of university students in Australia, only 16% reported high levels of confidence to manage their menstruation at university [16]. This was significantly associated with the quality of bathroom facilities. Supportive infrastructure is recognised as an integral requirement for menstrual health and hygiene, with comfort, privacy, safety, and access to water and soap for washing, and mirrors for checking for leakage all being highlighted as important in providing menstrual-friendly facilities [1, 25–28]. Audits of public toilets in New York City have highlighted many inadequacies for menstrual management, and significant consequences for those reliant on these services including homeless populations [29]. In many education institutions and workplace toilets, wash basins are placed outside the cubicles presenting a barrier to rinsing reusable products or hands when changing materials.

The burden of cleaning reusables was highlighted in some open text responses. While this view was endorsed by only 23% of current reusable product users in the survey, half of ever users and 65% of never users held this view. These different perceptions may suggest that cleaning difficulties were a reason for discontinued use of reusables and may present a disincentive to start using reusables for those who have not tried them. Greater visibility and information about using reusable products may support a realistic understanding of cleaning and support uptake. Similarly, many more participants using reusables agreed that these products were good protection and comfortable. More information capturing other users’ experiences may help further support uptake, as confidence and trust in the ability and effectiveness of reusable alternatives can be built.

## Strengths and limitations

Recruitment for the SDRR survey was conducted online using convenience sampling which may potentially result in sampling bias. Those with higher socioeconomic status are likely to be more represented in social media cross-sectional study recruitment as well as those who are more educated [30]. As cost and education were found to be associated with menstrual product choice, our results may overrepresent the prevalence of reusable use. As a cross-sectional study, data were collected at only one point in time, we are unable to infer the direction of effects and results may not represent long-term reusable use.

During the recruitment period, Victoria was under COVID-19 lockdown restrictions thus many participants may have been studying or working from home which might have impacted product choices. The survey was advertised as a general survey surrounding young people’s health and did not mention menstrual products. This reduced bias from individuals with an interest in the topic of menstrual choices from being more likely to participate.

Our open-text question asked participants to report if there was “anything else” they wanted to share about reusable products or product choices, which provided space for sharing a broad range of topics, it did not explicitly ask participant to identify barriers to use of reusables which may have resulted in different responses. Those providing open text responses may have different experiences, however this these data highlight areas for future inquiry, including difficulties learning to use menstrual cups comfortably, challenges identifying cups with the right ‘fit’, detailed information needs, and the design of facilities to support using reusable products outside the home.

## Conclusions

This study provides the first estimate of menstrual product preferences among young people in Australia. Almost half of participants had used reusable menstrual products. Potential barriers to uptake include information needs, the high upfront cost, difficulties using products such as menstrual cups and challenges of changing reusable products outside the home. Many young people highly valued environmental sustainability in selecting a menstrual product, and some noted benefits for comfort using reusable underwear or a menstrual cup. In addressing young people’s menstrual product needs, reusable options should be considered. Improving the quality of education about menstrual products may empower young people to select sustainable alternatives and improve experiences. Similarly, upgrading facilities such as bathrooms to improve comfort changing reusable products may enable greater reusable product use, including cost savings over time and environmental sustainability.

## Supplementary Information

**Additional file 1. Table S1.** Perceptions of reusable menstrual products by reusable product usage.

## Data Availability

The datasets generated during and analyzed during the current study are not publicly available due to participant privacy and confidentiality but are available from the corresponding author on reasonable request, pending approval from the Alfred Health Research Ethics Committee. Data requests can be made to Megan Lim via email at megan.lim@burnet.edu.au.

## References

[1] Hennegan J, Winkler IT, Bobel C, Keiser D, Hampton J, Larsson G (2021). Menstrual health: a definition for policy, practice, and research. Sex Reprod Health Matters.

[2] Sommer M, Sahin M (2013). Overcoming the taboo: advancing the global agenda for menstrual hygiene management for schoolgirls. Am J Public Health.

[3] UNICEF. Guide to menstrual hygiene materials. New York: UNICEF; 2019.

[4] Mahajan T. Imperfect Information in Menstrual Health and the Role of Informed Choice. Indian J Gender Stud. 2019:0971521518811169.

[5] Recyclopaedia, ACT Government. Menstrual Products and sustainability Auastralian Capital Teritory https://www.cityservices.act.gov.au/recyclopaedia/factsheets/menstrual-products: ACT Government; 2021 [

[6] Blair L, Bajón-Fernández Y, Villa R (2022). An exploratory study of the impact and potential of menstrual hygiene management waste in the UK. Cleaner Eng Technol.

[7] Peberdy E, Jones A, Green D (2019). A study into public awareness of the environmental impact of menstrual products and product choice. Sustainability.

[8] Hait A, Powers SE (2019). The value of reusable feminine hygiene products evaluated by comparative environmental life cycle assessment. Resour Conserv Recycl.

[9] Van Eijk AM, Zulaika G, Lenchner M, Mason L, Sivakami M, Nyothach E (2019). Menstrual cup use, leakage, acceptability, safety, and availability: a systematic review and meta-analysis. The Lancet Public Health.

[10] Van Eijk AM, Jayasinghe N, Zulaika G, Mason L, Sivakami M, Unger HW (2021). Exploring menstrual products: a systematic review and meta-analysis of reusable menstrual pads for public health internationally. PLoS ONE.

[11] Barrington DJ, Robinson HJ, Wilson E, Hennegan J (2021). Experiences of menstruation in high income countries: a systematic review, qualitative evidence synthesis and comparison to low- and middle-income countries. PLoS ONE.

[12] Munro AK, Hunter EC, Hossain SZ, Keep M (2021). A systematic review of the menstrual experiences of university students and the impacts on their education: a global perspective. PLoS ONE.

[13] Choi H, Lim N-K, Jung H, Kim O, Park H-Y (2021). Use of menstrual sanitary products in women of reproductive age: Korea Nurses’ Health Study. Osong Public Health Res Perspect.

[14] Medina-Perucha L, López-Jiménez T, Holst AS, Jacques-Aviñó C, Munrós-Feliu J, Martínez-Bueno C (2022). Use and perceptions on reusable and non-reusable menstrual products in Spain: a mixed-methods study. PLoS ONE.

[15] Free Pads and Tampons In All Government Schools [press release]. 2020.

[16] Munro AK, Keep M, Hunter EC, Hossain SZ (2022). Confidence to manage menstruation among university students in Australia: evidence from a cross-sectional survey. Womens Health.

[17] Coombe J, Bittleston H, Hocking JS (2022). Access to period products during the first nation-wide lockdown in Australia: results from an online survey. Women Health.

[18] Lim MSC, Agius PA, Carrotte ER, Vella AM, Hellard ME (2017). Young Australians' use of pornography and associations with sexual risk behaviours. Aust N Z J Public Health.

[19] Harris PA, Taylor R, Minor BL, Elliott V, Fernandez M, O'Neal L (2019). The REDCap consortium: building an international community of software platform partners. J Biomed Inform.

[20] Harris PA, Taylor R, Thielke R, Payne J, Gonzalez N, Conde JG (2009). A metadata-driven methodology and workflow process for providing translational research informatics support. J Biomed Inform.

[21] Hennegan J, Nansubuga A, Akullo A, Smith C, Schwab KJ (2020). The menstrual practices questionnaire (MPQ): development, elaboration, and implications for future research. Glob Health Action.

[22] Ritchie J, Lewis J, Nicholls CM, Ormston R (2014). Qualitative research practice: a guide for social science students & researchers.

[23] Hsieh H-F, Shannon SE (2005). Three approaches to qualitative content analysis. Qual Health Res.

[24] Curry C, Ferfolja T, Holmes K, Parry K, Sherry M, Armour M. Menstrual health education in Australian schools. Curriculum Studies in Health and Physical Education. 2022:1–14.

[25] Schmitt M, Clatworthy D, Ogello T, Sommer M (2018). Making the case for a female-friendly toilet. Water.

[26] UNICEF. Guidance on Menstrual Health and Hygiene. New York: UNICEF. Available from https://www.unicef.org/wash/files/UNICEF-Guidance-menstrual-health-hygiene-2019.pdf. Accessed July 2019; 2019.

[27] Hennegan J, Shannon AK, Rubli J, Schwab KJ, Melendez-Torres GJ (2019). Women's and girls' experiences of menstruation in low- and middle-income countries: a systematic review and qualitative metasynthesis. PLoS Med.

[28] Schmitt ML, Hagstrom C, Nowara A, Gruer C, Adenu-Mensah NE, Keeley K (2021). The intersection of menstruation, school and family: experiences of girls growing up in urban cities in the USA. Int J Adolesc Youth.

[29] Maroko AR, Hopper K, Gruer C, Jaffe M, Zhen E, Sommer M (2021). Public restrooms, periods, and people experiencing homelessness: an assessment of public toilets in high needs areas of Manhattan, New York. PLoS ONE.

[30] Eddy S, Douglass C, Raggatt M, Thomas A, Lim MSC. Trends in STI testing, sexual health knowledge and behaviours, and pornography use in cross-sectional samples of young people in Victoria, Australia; 2015–2021. under review. 10.1071/SH22122 36966731

