# Supplementary material for: Reusable period products: use and perceptions among young people in Victoria, Australia
Source: BMC Womens Health. 2023 Mar 11;23:102. doi: 10.1186/s12905-023-02197-3 (PMC10006563; doi:10.1186/s12905-023-02197-3)
Supplement: Supplementary file 1 — Additional file 1. Table S1. Perceptions of reusable menstrual products by reusable product usage. [file 12905_2023_2197_MOESM1_ESM.docx]

**Supplementary Materials**

**Table S1.** Perceptions of reusable menstrual products by reusable product usage

|  | Agree | | Disagree | | I don't wish to say | | PR (95% CI) |
| --- | --- | --- | --- | --- | --- | --- | --- |
| Statement | n | % | n | % | n | % |  |
| reusables are good for the environment |  |  |  |  |  |  |  |
| never | 278 | 89.68 | 26 | 8.39 | 6 | 1.94 | 1.00 |
| ever | 54 | 87.10 | 8 | 12.90 | 0 | 0.00 | 1.04 (0.96, 1.13) |
| current | 218 | 98.20 | 3 | 1.35 | 1 | 0.32 | 0.93 (0.90, 0.96) |
| reusables are low cost |  |  |  |  |  |  |  |
| never | 152 | 68.47 | 152 | 68.47 | 6 | 1.94 | 1.00 |
| ever | 45 | 72.58 | 17 | 27.42 | 0 | 0.00 | 0.85 (0.77, 0.93) |
| current | 140 | 63.06 | 82 | 36.94 | 0 | 0.00 | 0.91 (0.86, 0.97) |
| Reusables are comfortable |  |  |  |  |  |  |  |
| never | 150 | 48.39 | 118 | 38.06 | 42 | 13.55 | 1.00 |
| ever | 32 | 51.61 | 29 | 46.77 | 1 | 0.32 | 1.02 (0.93, 1.13) |
| current | 195 | 87.84 | 24 | 10.81 | 3 | 0.97 | 0.77 (0.73, 0.81) |
| Reusables are good protection from leakage |  |  |  |  |  |  |  |
| never | 182 | 58.71 | 86 | 27.74 | 42 | 13.55 | 1.00 |
| ever | 34 | 54.84 | 23 | 37.10 | 5 | 1.61 | 1.06 (0.96, 1.17) |
| current | 189 | 85.14 | 30 | 13.51 | 3 | 0.97 | 0.86 (0.81, 0.91) |
| Reusables are difficult to change outside of home |  |  |  |  |  |  |  |
| never | 243 | 78.39 | 50 | 16.13 | 17 | 5.48 | 1.00 |
| ever | 45 | 72.58 | 14 | 22.58 | 3 | 0.97 | 1.06 (0.96, 1.16) |
| current | 156 | 70.27 | 65 | 29.28 | 1 | 0.32 | 1.11 (1.04, 1.17) |
| Reusables are unhygienic/dirty/gross |  |  |  |  |  |  |  |
| never | 92 | 29.68 | 205 | 66.13 | 13 | 4.19 | 1.00 |
| ever | 16 | 25.81 | 45 | 72.58 | 1 | 0.32 | 1.03 (0.96, 1.10) |
| current | 15 | 6.76 | 205 | 92.34 | 2 | 0.65 | 1.14 (1.10, 1.18) |
| Reusables are too much effort to clean |  |  |  |  |  |  |  |
| never | 201 | 64.84 | 92 | 29.68 | 17 | 5.48 | 1.00 |
| ever | 30 | 48.39 | 31 | 50.00 | 1 | 0.32 | 1.15 (1.05, 1.26) |
| current | 50 | 22.52 | 170 | 76.58 | 2 | 0.65 | 1.35 (1.28, 1.42) |
